# Immunophenotypic and Molecular Features of Acute Myeloid Leukemia with Plasmacytoid Dendritic Cell Differentiation Are Distinct from Blastic Plasmacytoid Dendritic Cell Neoplasm

**DOI:** 10.3390/cancers14143375

**Published:** 2022-07-11

**Authors:** Wei Wang, Jie Xu, Joseph D. Khoury, Naveen Pemmaraju, Hong Fang, Roberto N. Miranda, C. Cameron Yin, Siba El Hussein, Fuli Jia, Zhenya Tang, Shimin Hu, Marina Konopleva, L. Jeffrey Medeiros, Sa A. Wang

**Affiliations:** 1 Department of Hematopathology, The University of Texas MD Anderson Cancer Center, Houston, TX 77030, USA; jxu9@mdanderson.org (J.X.); jkhoury@unmc.edu (J.D.K.); hfang@mdanderson.org (H.F.); roberto.miranda@mdanderson.org (R.N.M.); cyin@mdanderson.org (C.C.Y.); siba_elhussein@urmc.rochester.edu (S.E.H.); fjia@mdanderson.org (F.J.); ztang@mdanderson.org (Z.T.); shu1@mdanderson.org (S.H.); ljmedeiros@mdanderson.org (L.J.M.); 2 Department of Leukemia, The University of Texas MD Anderson Cancer Center, Houston, TX 77030, USA; npemmaraju@mdanderson.org (N.P.); mkonople@mdanderson.org (M.K.)

**Keywords:** acute myeloid leukemia, BPDCN, plasmacytoid dendritic cells, immunophenotype, flow cytometry, mutation

## Abstract

**Simple Summary:**

Acute myeloid leukemia with plasmacytoid dendritic cell differentiation (pDC-AML) and blastic plasmacytoid dendritic cell neoplasm (BPDCN) are two leukemias characterized by neoplastic pDC proliferation. In this study, we aimed to explore the immunophenotypic and molecular profiles of pDC-AML and compare them with BPDCN. We found that pDCs in pDC-AML and BPDCN possessed different immunophenotype. The mutation profiles of these two were also different. These findings demonstrate that pDC-AML and BPDCN are two distinct entities and pDCs in these two entities derive from different subsets of pDC precursors.

**Abstract:**

Acute myeloid leukemia (AML) with ≥2% plasmacytoid dendritic cells (pDC) has been recently described as AML with pDC differentiation (pDC-AML) characterized by pDC expansion with frequent *RUNX1* mutations. In this study, we investigated a cohort of 53 pDC-AML cases representing about 3% of all AML cases. We characterized their immunophenotype and genetic profiles and compared these findings with blastic plasmacytoid dendritic cell neoplasm (BPDCN). pDC-differentiation/expansion was preferentially observed in AML with an immature myeloid or myelomonocytic immunophenotype, where myeloblasts were frequently positive for CD34 (98%), CD117 (94%), HLA-DR (100%) and TdT (79%), with increased CD123 (89%) expression. The median number of pDCs in pDC-AML was 6.6% (range, 2% to 26.3%) and their immunophenotype reminiscent of pDCs in early or intermediate stages of differentiation. The immunophenotype of pDCs in pDC-AML was different from BPDCN (*n* = 39), with major disparities in CD34 (96% vs. 0%), CD56 (8% vs. 97%) and TCL1 (12% vs. 98%) and significant differences in frequency of CD4, CD13, CD22, CD25, CD36, CD38, CD117 and CD303 expression. At the molecular level, the genetic landscapes of pDC-AML and BPDCN also differ, with *RUNX1* mutations detected in 64% of pDC-AML versus 2% of BPDCN. Disparities in *TET2* (21% vs. 56%), *FLT3* (23% vs. 0%), *DNMT3A* (32% vs. 10%) and *ZRSR2* (2% vs. 16%) (all *p* < 0.05) were also detected. The distinct immunophenotypic and mutation profiles of pDC-AML and BPDCN indicate that the neoplastic pDCs in pDC-AML and BPDCN derived from different subsets of pDC precursors.

## 1. Introduction

Plasmacytoid dendritic cells (pDCs) are produced in the bone marrow and traffic to the peripheral tissues and organs when they mature [1]. There are various subsets of pDCs with different functions: some recognize pathogen-derived nucleic acids and respond rapidly by producing massive type I interferon, while others lack interferon production capacity but are involved in antigen presentation [1]. The heterogeneity of pDCs may be attributable, at least in part, to diverse progenitor lineages [2]. The origin and development of pDCs as well as their relationship to classic myeloid and lymphoid cells have been extensively studied with some controversial results. Previous studies indicated that pDCs can be derived from either a common myeloid dendritic cell progenitor or a common lymphoid progenitor [2,3,4]. Cells from myeloid and lymphoid progenitors differentiate and converge into a single phenotypically similar pDC population. Although showing similar morphologic and immunophenotypic features, myeloid- and lymphoid-derived pDCs have been shown to be transcriptionally and functionally heterogeneous [2]. However, a very recent study challenged the idea of the lymphoid origin of pDCs and demonstrated that pDCs and conventional dendritic cells (cDCs) derived from the same progenitors. pDCs are clonally related to the cCD1 subset of cDCs, supporting the classic definition of pDCs as a part of the DC lineage [5].

Mirroring the heterogeneity of normal pDCs, neoplastic proliferations of pDCs can manifest with different disease types such as blastic plasmacytoid dendritic cell neoplasm (BPDCN), mature pDC proliferations associated with myeloid neoplasms and recently described AML with pDC expansion/differentiation (pDC-AML) [6,7,8,9]. Patients with pDC-AML have been shown to have an inferior prognosis when compared with AML patients without pDC expansion [6]. *RUNX1* mutations are common in pDC-AML, and the same gene mutations and cytogenetic abnormalities have been shown in pDC and myeloblasts, indicating their shared cell of origin. The immunophenotype of pDCs in pDC-AML was explored by others in a few studies [6,7,8,10] with variable results reported; CD34, TdT and CD56 were positive in 61%, 40% and 13% of cases, respectively, in one study [6] compared to 33%, 11% and 0% of cases, respectively, in another report [8].

In this study, we presented 53 cases of pDC-AML from our cancer center, the largest cohort to date. We comprehensively explored the immunophenotypic and molecular features of pDC-AML and discussed the differential diagnosis with BPDCN.

## 2. Materials and Methods

### 2.1. Study Cohort

We retrospectively screened cases with a diagnosis of AML and mixed phenotype acute leukemia (MPAL) including myeloid/T or myeloid/B, from 7 January 2017 through 31 December 2020 that underwent flow cytometric immunophenotypic analysis; 1768 cases were identified. Among this group, 53 cases showed increased pDCs defined as ≥2% of total nucleated cells by flow cytometry [6]. Therefore, this patient subset represents about 3% of all AML or MPAL cases diagnosed or referred during the same time period. Corresponding clinical and laboratory data were obtained through electronic medical chart review. For immunophenotypic and molecular comparison, BPDCN cases were collected and their detailed clinicopathological features have been described previously [11,12]. This study was approved by the Institutional Review Board at our institution.

### 2.2. Immunophenotypic Analysis by Flow Cytometry

Flow cytometry immunophenotyping was performed on fresh bone marrow aspirate specimens using a standard staining and lyse/wash technique (PharmLyseTM, BD Biosciences, San Diego, CA, USA) as part of routine clinical work-up. Samples were examined with antibody panels designed to assess leukemic cells including: CD2, surface and cytoplasmic CD3, CD4, CD5, CD7, CD13, CD14, CD15, CD19, CD25, CD33, CD34, CD36, CD38, CD41, CD45, CD56, CD64, CD117, CD123, HLA-DR, MPO and TdT. An add-on panel for BPDCN was also performed in some cases consisting of CD2, CD4, CD7, CD34, CD38, CD45, CD56, CD64, CD123, CD303 and HLA-DR. For each analysis, a minimum of 200,000 events was acquired on CANTO II instruments (BD Biosciences), in which standardization was maintained using CS&T Beads with emphasis on comparable cross-platform performance. Data were analyzed using FCS Express (De novo Software, Glendale, CA, USA).

For the patterns and intensity assessed by flow cytometry, positivity for most markers was defined as expression in ≥20% of cells using the cutoff set by comparison to background fluorescence, with 20%–80% of cells being partially positive and >80% of cells being uniformly positive. A small subset was defined as expression in <20% of cells, but expression was deemed as clearly separated from the negative population.

### 2.3. Immunophenotypic Analysis by Immunohistochemistry

CD123/TCF4 and TCL1 immunohistochemistry was performed following a protocol described previously [13].

### 2.4. Next Generation Sequencing Analysis

Targeted next-generation sequencing methods were used to detect genes commonly mutated in myeloid neoplasms. Among 53 pDC-AML cases, 51 were tested using a panel including 81 genes (*ANKRD26*, *ASXL1*, *ASXL2*, * BCOR*, * BCORL1*, * BRAF*, * BRINP3*, * CALR*, * CBL*, * CBLB*, * CBLC*, * CEBPA*, * CREBBP*, * CRLF2*, * CSF3R*, * CUX1*, * DDX41*, * DNMT3A*, * EED*, * ELANE*, * ETNK1*, * ETV6*, * EZH2*, * FBXW7*, * FLT3*, * GATA1*, * GATA2*, * GFI1*, * GNAS*, * HNRNPK*, * HRAS*, * IDH1*, * IDH2*, * IKZF1*, * IL2RG*, * IL7R*, * JAK1*, * JAK2*, * JAK3*, * KDM6A*, * KIT*, * KMT2A*, * KRAS*, * MAP2K1*, * MPL*, * NF1*, * NOTCH1*, * NPM1*, * NRAS*, * PAX5*, * PHF6*, * PIGA*, * PML*, * PRPF40B*, * PTEN*, * PTPN11*, * RAD21*, * RARA*, * RUNX1*, * SETBP1*, * SF1*, * SF3A1*, * SF3B1*, * SH2B3*, * SMC1A*, * SMC3*, * SRSF2*, * STAG1*, * STAG2*, * STAT3*, * STAT5A*, * STAT5B*, * SUZ12*, * TERC*, * TERT*, * TET2*, * TP53*, * U2AF1*, * U2AF2*, *WT1* and *ZRSR2*). The remaining 2 cases were tested using a panel including 28 genes (*ABL1*, * ASXL1*, * BRAF*, * DNMT3A*, * EGFR*, * EZH2*, * FLT3*, * GATA1*, * GATA2*, * HRAS*, * IDH1*, * IDH2*, * IKZF2*, * JAK2*, * KIT*, * KMT2A*, * KRAS*, * MDM2*, * MPL*, * MYD88*, * NOTCH1*, * NPM1*, * NRAS*, * PTPN11*, * RUNX1*, * TET2*, *TP53* and *WT1*) [14].

### 2.5. Statistical Analysis

Chi-square was used to assess categorical variables. A *p*-value of <0.05 was considered statistically significant. Descriptive analyses and other statistical analyses were performed using GraphPad Prism 9 (La Jolla, CA, USA).

## 3. Results

### 3.1. Clinicopathologic Characteristics

Our cohort was composed of 53 cases of pDC-AML. There were 36 men and 17 women with a median age of 69 years (range, 24–90). Fourteen (26%) cases of pDC-AML showed monocytic differentiation including 4 AML with *CBFB* rearrangement and 10 acute myelomonocytic leukemia. There were 3 cases of MPAL including 2 mixed myeloid/B and 1 mixed myeloid/T. In both cases of MPAL-myeloid/B, myeloblasts were predominant and B lymphoblasts were a minor component, representing 5% and 18% of total blasts. In the case of MPAL-myeloid/T, blasts were positive for multiple myeloid markers (CD13, CD117 and MPO) as well as CD7 and cytoplasmic CD3.

By flow cytometry, the percentages of pDCs in total nucleated cells ranged from 2% to 26.3% with a median of 6.6% in these 53 cases.

### 3.2. Histopathologic Features of pDCs in pDC-AML

**Figure 1:**
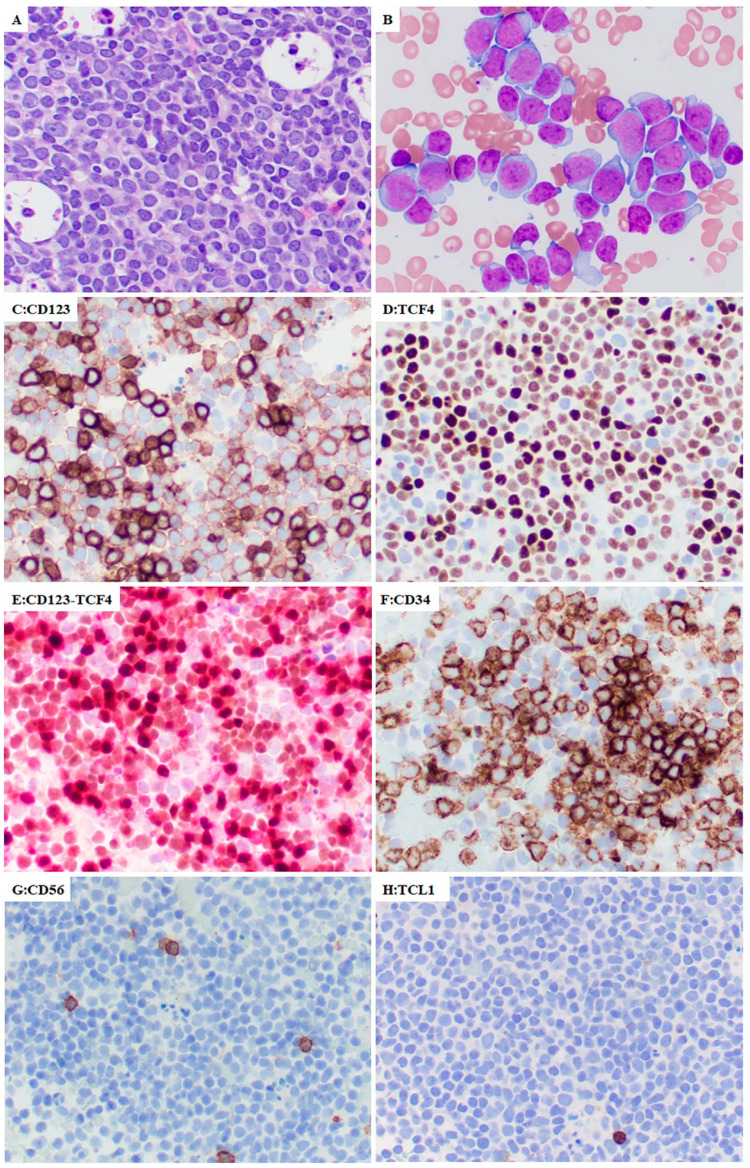
The morphology and immunophenotype (immunohistochemistry) of pDC-AML. (**A**). Diffuse infiltrate of blasts with variable size on bone marrow core biopsy. (**B**). Bone marrow aspirate smear shows two types of cells. One is large with round to slightly irregular nuclei, dispersed chromatin and small to moderate amounts of basophilic cytoplasm, consistent with myeloblasts. Another is smaller and many have dispersed chromatin and cytoplasmic pseudopods, consistent with pDCs. By immunohistochemistry, pDCs are strongly positive for CD123 (**C**,**E**) and TCF4 (**D**,**E**), and weakly positive for CD34 (**F**). In contrast, myeloblasts are weakly positive for CD123 (**C**,**E**) and TCF4 (**D**,**E**) with strong expression of CD34 (**F**). Both pDCs and myeloblasts are negative for CD56 (**G**) and TCL1 (**H**) in this case.

Morphology and bone marrow distribution of pDC were assessed in conjunction with CD123/TCF4 immunohistochemistry in 18 cases in which a minimum of 10% pDCs was detected by flow cytometry. All cases showed an interstitial distribution of pDCs intermixed with leukemic blasts (Figure 1A,C) on the core biopsy. In cases with higher percentages of pDCs, in addition to an interstitial distribution, pDCs were also present in loose clusters. A nodular pattern, which is often seen in mature pDC proliferations associated with myeloid neoplasms, was not common in pDC-AML and detected in only 1 case, in which a few small pDCs nodules were present in addition to interstitially distributed pDCs.

On bone marrow aspirate smears, cells with pDC morphology were of medium size with oval to irregular nuclei, dispersed chromatin with frequent small distinct nucleoli, and scant to moderate basophilic cytoplasm polarized at one side with the appearance of pseudopodia (Figure 1B). 

### 3.3. Immunophenotype of pDCs in pDC-AML

The immunophenotype of pDCs was assessed in reference to normal/reactive pDCs [10,11,15] in bone marrow; normal pDCs are brightly positive for CD123 and HLA-DR and show a maturation pattern with progressive loss of CD34 and CD117, and gradual acquisition of CD4, CD36 and CD303. Normal pDCs are negative for CD3, CD14, CD15, CD19 and MPO with a tiny subset positive for CD56.

**Figure 2:**
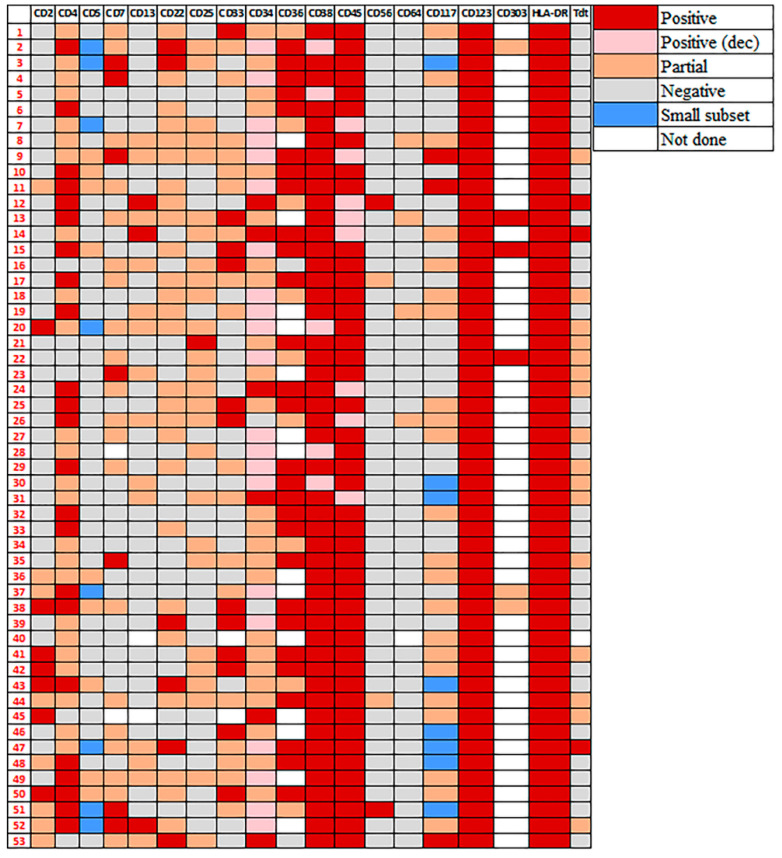
The immunophenotype of pDCs in pDC-AML by flow cytometry. The detailed immunophenotype of 53 cases of pDC-AML is displayed (cases # 1–34, *RUNX1* mutated; cases # 35–53, *RUNX1* wild type). For the category of positive (dec), CD34 expression was compared to the coexisting myeloblasts; CD38 and CD45 expression was compared to normal/reactive pDCs. Of note, similar to positive category, positive (dec) cases also showed >80% cells being positive.

**Figure 3:**
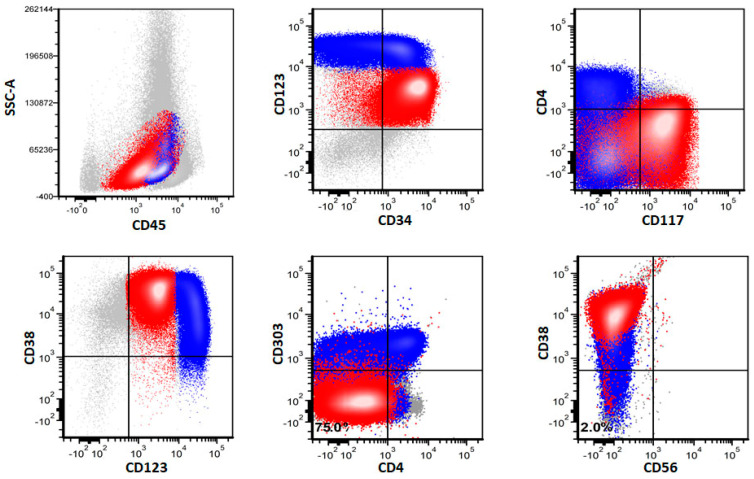
A representative case of pDC-AML by flow cytometry. Myeloblasts are highlighted in red and pDCs are highlighted in blue.

The detailed immunophenotype of pDCs in pDC-AML is illustrated in Figure 2 (cases # 1–34, *RUNX1* mutated; cases # 35–53, *RUNX1* wild type) and a representative case of AML-pDC is shown in Figure 3 to illustrate the immunophenotype of pDCs and myeloblasts. Similar to normal/reactive pDCs, the pDCs in all 53 pDC-AML were brightly positive for CD123 and HLA-DR. CD45 was expressed at a level similar to normal pDCs in 45 (85%) and was decreased in 8 (15%) cases. CD38 was positive in all cases, bright in 48 (91%) and decreased in 5 (9%) cases. CD34 was positive in 96% (51/53) of cases (27 uniform and 24 partial). When compared to the coexisting myeloblasts, 45 (88%) positive cases showed a lower CD34 expression in pDCs. Other markers frequently positive included CD303 (6/6, 100%, 3 uniform and 3 partial), CD36 (37/39, 95%, 28 uniform and 9 partial), CD4 (47/53, 89%, 23 uniform and 24 partial), CD22 (32/53, 60%, 6 uniform and 26 partial), CD33 (30/52, 59%, 12 uniform and 18 partial), CD7 (27/51, 53%, 7 uniform and 20 partial), CD25 (27/53, 51%, 1 uniform and 26 partial) and CD117 ( 25/53, 47%, 3 uniform and 22 partial). Markers that were positive but less common included: CD13 (17/52, 33%, 3 uniform and 14 partial), CD2 (15/53, 28%, 7 uniform and 8 partial) and CD5 (9/53, 17%, all partial; but also shown on a small subset in additional 8, 15%). CD56 expression was only observed in 4 (8%) cases with 2 partial and 2 uniform. Of note, the myeloblasts in the latter two cases were also uniformly positive for CD56. TdT was positive in 19/52 (37%) cases (16 partial and 3 uniform) and myeloblasts in all these 19 cases also showed TdT expression. CD64 was expressed in 4/52 (8%) cases (all with partial expression). Thirteen cases were evaluated for CD71: 6 were negative and 7 were positive (3 partial and 4 uniform). Partial cytoplasmic CD3 was observed in 1 (2%) case. pDCs in all cases assessed were negative for CD14 (*n* = 53), CD15 (*n* = 53), CD19 (*n* = 53) and MPO (*n* = 52).

**Figure 4:**
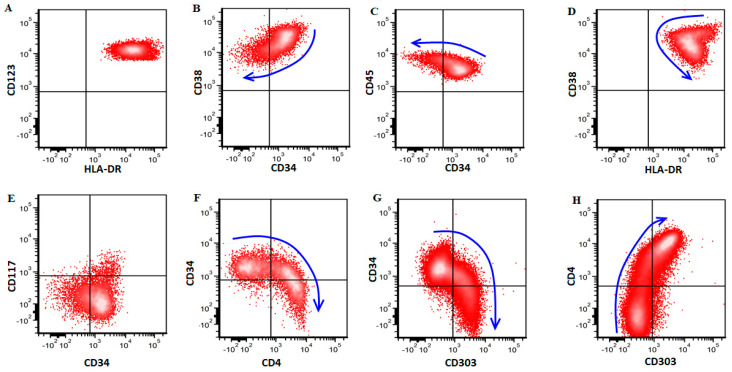
The maturation pattern of pDCs in a case of pDC-AML. pDCs are brightly positive for CD123 (**A**) and HLA-DR (**D**). The earliest pDCs are positive for CD34 (**B**,**C**,**F**,**G**), CD117 (dim, **E**), CD38 (bright, **B**), negative for CD4 (**F**,**H**) and CD303 (**G**,**H**). As they mature, the expression of CD34, CD38 and CD117 decreases, and the expression of CD4 and CD303 increases (**H**).

As noted above, except for uniformly bright CD123 (Figure 4A) and bright, mildly variable HLA-DR expression (Figure 4D), most antigens on pDCs in pDC-AML were expressed in a heterogeneous manner retaining a degree of differentiation/maturation reminiscent of normal/reactive pDCs in bone marrow; the brightest CD34+ subset corresponded to the earliest pDCs that were dim to negative for CD4 and CD303 (Figure 4F–H). These cells were also brightly positive for CD38 (Figure 4B) with dimmer CD45 (Figure 4C). Accordingly, CD34-dim to negative subsets corresponded to later stages of pDC maturation that were positive for CD4 and CD303, with decreased CD38 and increased CD45. CD117, when positive, also highlighted very early pDCs (Figure 4E).

The comparison between *RUNX1* mutated (# 1–34, Figure 2) and *RUNX1* wild type (# 35–53, Figure 2) cases were performed; the median percentage of pDC by flow cytometry was 7.3% (range, 2.0–26.3) in the *RUNX1* mutated cases, not significantly different from 4.3% (range, 2.2–20.3) of the *RUNX1* wild type group (*p* = 0.19). However, CD2 expression by pDCs was conspicuously more frequent in *RUNX1* wild type cases (13/19, 68% versus 2/34, 5.9%; *p* < 0.001) (Figure 2). The expression pattern of other markers showed no significant difference among these two groups.

### 3.4. Immunophenotypic Differences of pDCs in pDC-AML and BPDCN

We compared the immunophenotype of pDCs in pDC-AML to neoplastic pDCs in 39 BPDCN cases in which comprehensive flow cytometric analysis was performed. As shown in Table 1, CD34 was positive in 96% of pDC-AML cases but none in BPDCN (*p* < 0.0001). In contrast, CD56 was positive in only 8% of pDCs in pDC-AMLs compared to 97% of BPDCN cases positive for CD56 (*p* < 0.0001). CD303, a specific marker for pDCs, was positive in all pDC-AML cases and only 44% of BPDCN cases were positive (*p* = 0.046). Of note, the number of pDC-AML cases tested for CD303 in our study was rather small (*n* = 6). Two myeloid markers CD13 and CD117 were significantly higher in pDC-AML cases, 33% and 47%, respectively, compared to to 0% (*p* = 0.0001) and 9% (*p* = 0.0001) in BPDCN. CD22 and CD25 expression was seen in 60% and 51% of pDC-AML cases, respectively, whereas none of BPDCN cases expressed these two markers (*p* < 0.0001). CD36 and CD38 expression was more frequently positive in pDC-AML than BPDCN (Table 1) and on the contrary, CD4 expression was more frequently seen in BPDCN (*p* = 0.04).

### 3.5. Immunophenotype of Myeloblasts in pDC-AML by Flow Cytometry

The myeloblasts in pDC-AML were positive for CD34 in 52 (98%) of 53 cases. CD117 was positive in 50 (94%) of 53 cases. All 53 cases were positive for HLA-DR. Similarly, all 53 cases were positive for CD123 and 47 (89%) showed an increased (compared to normal myeloid precursors) and uniform CD123 expression. In total, the immunophenotype of CD34+/CD117+/HLA-DR+/CD123+inc of myeloblasts was observed in 43 (81%) of 53 pDC-AML cases. TdT was positive in 42 (79%) of 53 cases. MPO was positive in 26 (49%) of 53 cases. Of note, although myeloblasts in most cases showed increased CD123 expression, their expression level was still lower than pDCs from the same sample (Figure 3).

Immunophenotypic comparison of myeloblasts between *RUNX1* mutated and wild type cases revealed that cases with mutated *RUNX1* cases had more frequent increased CD123 expression than cases with wild type *RUNX1* (97%, 33/34 versus 74%, 14/19; *p* = 0.010).

### 3.6. Immunophenotype of pDCs and Myeloblasts in pDC-AML by Immunohistochemistry

Immunostains were performed in 17 cases; CD123 and TCF4 as well as CD123/TCF4 double stain highlighted increased pDCs in all cases and the expression of both CD123 and TCF4 was strong in pDCs. Interestingly, in addition to pDCs, a subset of myeloblasts also showed CD123 and TCF4 expression but their expression level was much weaker than pDCs (Figure 1). TCL1 was positive in pDCs in 2 (12%) of 17 cases, and negative in myeloblasts in all 17 cases.

### 3.7. Molecular Profile of pDC-AML

**Figure 5:**
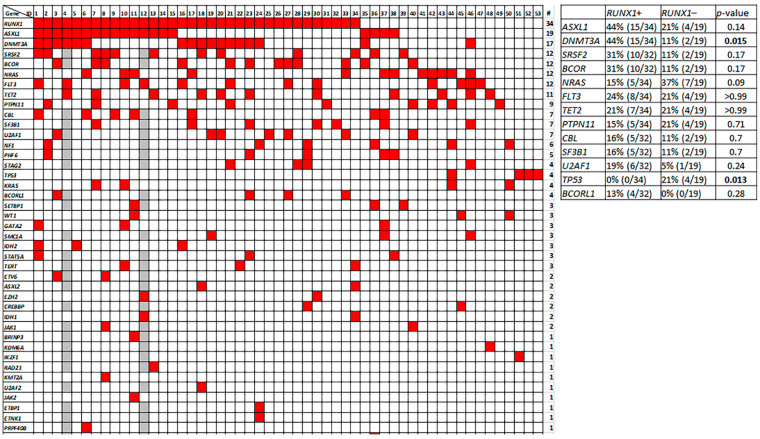
The mutational profile of pDC-AML. Cases # 1–34 with *RUNX1* mutation, and cases # 35–53 with wild type *RUNX1*. The case number in this figure correlates with the case number assigned in Figure 1.

The detailed mutation profile is illustrated in Figure 5. *RUNX1* was the most frequently mutated gene, detected in 34 (64%) cases, followed by *ASXL1* in 19 (36%), *DNMT3A* in 17 (32%), *SRSF2* and *BCOR* each in 12/51 (24%). *NRAS* and *FLT3* mutations were detected in 12 (23%) patients each. For *FLT3*, internal tandem duplication (ITD) was detected in 4 cases, D835 mutation in 4 cases, Y842C mutation in 2 cases, V592A mutation in 1 case and an in-frame deletion-insertion mutation (Y591_V592delinsH) in 1 case.

All 34 *RUNX1* mutated cases had other co-mutations detected; *ASXL1* and *DNMT3A* were most frequent, seen in 15 (44%) cases each. Six (18%) showed *RUNX1*, *ASXL1* and *DNMT3A* mutations simultaneously.

When we divided cases into *RUNX1* mutated (*n* = 34) versus *RUNX1* wild type (*n* = 19) subgroups, *DNMT3A* mutations were more common in the *RUNX1* mutated subgroup (44% versus 11%, *p* = 0.015), whereas *TP53* mutations were only detected in the *RUNX1* wild type subgroup (4/19, 21% vs. 0/34, *p* = 0.013). There was no significant differences in the frequency of other mutated genes between these two groups.

### 3.8. Genetic Differences between pDC-AML and BPDCN

We compared the mutation profile of pDC-AML with 50 cases of BPDCN [12] in which NGS-based mutation analysis was performed. As shown in Table 2, compared to 64% of pDC-AML cases carrying *RUNX1* mutations, only 1 (2%) case of BPDCN showed *RUNX1* mutation (*p* < 0.0001). *TET2* mutations were the most common mutations in BPDCN, seen in 28/50 (56%) of cases. In contrast, only 11 (21%) pDC-AML cases showed *TET2* mutations (*p* = 0.0003). *FLT3* mutations were detected in 12 (23%) pDC-AML cases whereas no BPDCN case showed this mutation (*p* = 0.0003). *DNMT3A* mutations were detected more frequently in pDC-AML than BPDCN (32% versus 10%, *p* = 0.0079), and *ZRSR2* mutations occurred less frequently in pDC-AML than BPDCN (2% versus 16%, *p* = 0.03).

## 4. Discussion

In this study, we explored the immunophenotypic and molecular features of pDC-AML and compared these findings with those of blastic plasmacytoid dendritic neoplasm (BPDCN). Our data provide new insights into understanding the cell origin and characteristics of the neoplastic pDC proliferations associated with AML and BPDCN.

Using the same 2% cutoff of pDCs as used by others, the frequency of pDC-AML was about 3% in our study, slightly lower than the previously reported occurrence rate [6], which is likely due to the differences in patient demographics and variable subtypes of AML included in the study cohorts. pDC differentiation/expansion occurred preferentially in cases of AML with an immature myeloid immunophenotype or cases with myelomonocytic differentiation where the myeloblasts were frequently positive for CD34, CD117, HLA-DR and TdT. The pDCs were easily distinguishable from myeloblasts by their bright CD123 and HLA-DR expression detected by flow cytometry immunophenotyping and strong reactivity for the transcription factor TCF4 by immunohistochemistry. pDCs also had discernable morphologic features on BM aspirate smears. However, despite these differences from myeloblasts, pDCs exhibited a differentiation pattern that is part of a continuum with myeloblasts sharing expression of a number of markers such as CD34, CD117, CD123, CD13 and TdT in many cases. The myeloblasts of pDC-AML also showed significantly upregulated CD123 and TCF4 expression, indicating their potential relationship to pDCs. We also showed in BM biopsy specimens that pDCs associated with AML are mostly intermingled with myeloblasts, rarely forming large pDC nodules [16,17], the latter having been described in chronic myelomonocytic leukemia (CMML) or myeloproliferative neoplasms. The morphologic and immunophenotypic features of pDCs in pDC-AML support a shared clonal origin between pDCs and myeloblasts, as has been shown by others who performed molecular genetic studies on sorted cells [6,8].

NGS studies were performed in all pDC-AML cases in our study and *RUNX1* mutations were detected in 64%, a frequency slightly lower than 73% and 78% reported in two earlier reports [6,8]. Mutations in other genes were also detected in this cohort at frequencies ranging from 21%–36%, including *ASXL1*, * DNMT3A*, * SRSF2*, * BCOR*, * TET2*, * NRAS* and *FLT3*. The mutation profile of pDC-AML is distinct from BPDCN cases which showed infrequent to absent mutations of *RUNX1*, * DNMT3A*, * BCOR* and *FLT3*, but mutations of *TET2* in over half of cases. The immunophenotype of pDCs in pDC-AML was also distinct from BPDCN, with the most remarkable disparities in the frequency of CD34, CD56 and TCL1 expression. pDCs in pDC-AML are uniformly or partially positive for CD34, and mostly negative for TCL1 and CD56; in contrast, BPDCN was completely negative for CD34 and frequently positive for CD56 and TCL1 (Table 1). When compared to normal/reactive mature pDCs, the major immunophenotypic differences observed in pDCs of pDC-AML include altered (increased or decreased) expression of normal pDC markers, such as CD2, CD4, CD7, CD22, CD33, CD34, CD36 and TCL1, or expression of myeloid antigens such as CD117 and CD13. We showed that the pDCs in pDC-AML cases, although expanded and immunophenotypically atypical, appear to maintain a degree of differentiation/maturation (Figure 4), reminiscent of early and intermediate maturation stage pDCs in the bone marrow [10,11,15].

pDCs are heterogeneous and their cell of origin has been a focus of research interest in recent years. A previous study using mouse tissue suggested that pDCs are solely derived from lymphoid progenitors [18]; however, a recent study demonstrated that pDCs originate from the same progenitors as cDCs, which argues against their lymphoid origin [5]. The mutation profile and immunophenotypic differences between pDC-AML and BPDCN suggest that they may have derived from different pDC precursors. As noted above, pDCs in pDC-AML are derived from CD34-positive blasts; in contrast, pDCs in BPDCN are believed to be derived from the CD56+ subset of pDC precursors [19,20]. A myeloid origin of BPDCN has been supported by a frequent association of BPDCN with myelodysplastic syndromes (MDS), CMML and MPN [21,22,23,24], and the recent demonstration of a shared clonal origin of BPDCN with CMML [24,25,26,27].

The mechanisms behind pDC expansion/differentiation in pDC-AML were explored by Xiao et al. [6] who showed that in *RUNX1* mutated AML, the pDC transcriptional program is upregulated, promoting pDC differentiation and expansion. The same mechanisms likely contribute to elevated CD123 and TCF4 expression by myeloblasts in *RUNX1* mutated AML cases. However, one third of pDC-AML cases in our study did not carry *RUNX1* mutations, and these *RUNX1* wild type cases showed significantly more frequent *TP53* mutations, less *DNMT3A* mutations and conspicuously more frequent CD2 expression on pDCs (68%). The molecular genetic events contributing to pDC expansion/differentiation in *RUNX1* wild type pDC-AML need to be further studied.

CD123 targeted therapy has become a frontline therapy for patients with BPDCN given their high level of CD123 expression [13]. Anti-CD123 targeted therapy had been shown to reduce pDCs and leukemic blasts in a mouse model [6]. In pDC-AML, CD123 expression is increased in both pDCs and myeloblasts. We suggest to consider it as a distinct subtype of AML that may potentially benefit from CD123 targeted therapy.

## 5. Conclusions

pDC-AML is a rare subtype of AML and shows an immunophenotypic and mutation profile that differs greatly from BPDCN, suggesting different origin. The high expression of CD123 in both pDCs and myeloblasts in pDC-AML can be targeted for therapeutic purpose.

## Figures and Tables

**Table 1:** The immunophenotype of pDCs in pDC-AML (*n* = 53) and BPDCN (*n* = 39).

Markers	Positivity (%)		*p* Value
	pDC-AML	BPDCN	
CD2	28% (15/53)	19% (5/27)	0.42
cCD3	1.9% (1/53)	0% (0/15)	>0.99
**CD4**	89% (47/53)	100% (38/38)	**0.039**
CD5 *	17% (9/53)	3% (1/30)	0.086
CD7	53% (27/51)	64% (21/33)	0.37
**CD13**	33% (17/51)	0% (0/30)	**0.0001**
CD14	0% (0/53)	3% (1/34)	0.39
CD15	0% (0/53)	0% (0/28)	>0.99
CD19	0% (0/53)	0% (0/30)	>0.99
**CD22**	60% (32/53)	0% (0/26)	**<0.0001**
**CD25**	51% (27/53)	0% (0/22)	**<0.0001**
CD33	59% (30/51)	48% (16/33)	0.38
**CD34**	96% (51/53)	0% (0/27)	**<0.0001**
**CD36**	95% (37/39)	57% (17/30)	**0.0002**
**CD38**	100% (53/53)	88% (30/34)	**0.021**
CD45	100% (53/53)	100% (39/39)	>0.99
**CD56**	8% (4/53)	97% (36/37)	**<0.0001**
CD64	8% (4/52)	0% (0/36)	0.14
CD117 *	47% (25/53)	9% (3/34)	**0.0001**
CD123	100% (53/53)	100% (36/36)	>0.99
**CD303**	100% (6/6)	44% (7/16)	**0.046**
HLA-DR	100% (53/53)	100% (36/36)	>0.99
MPO	0% (0/52)	0% (0/16)	>0.99
TdT	37% (19/52)	25% (4/16)	0.55
TCL1 *	12% (2/17)	98% (46/47)	**<0.0001**

Note: CD5 *: in addition to 17% cases being positive, 15% (8/53) cases showed positivity in a small subset of cells in pDC-AML. CD117 *: in addition to 47% cases being positive, 15% (8/53) cases were positive in a small subset of cells in pDC-AML. TCL1 *: TCL1 expression was evaluated by immunohistochemistry and all other markers in the table were evaluated by flow cytometry. Bold markers and *p* values indicate a significance difference between these two groups.

**Table 2:** Comparison of the mutational profile between pDC-AML (*n* = 53) and BPDCN (*n* = 50).

	pDC-AML	BPDCN	*p* Value
*RUNX1*	64% (34/53)	2% (1/50)	**<0.0001**
*ASXL1*	36% (19/53)	46% (23/50)	0.32
*DNMT3A*	32% (17/53)	10% (5/50)	**0.0079**
*SRSF2*	24% (12/51)	9% (3/32)	0.14
*BCOR*	24% (12/51)	0% (0/32)	**0.0026**
*NRAS*	23% (12/53)	10% (5/50)	0.11
*FLT3*	23% (12/53)	0% (0/50)	**0.0003**
*TET2*	21% (11/53)	56% (28/50)	**0.0003**
*ZRSR2*	2% (1/51)	16% (5/32)	**0.030**
*IKZF1*	2% (1/51)	9% (3/32)	0.29

Bold markers and *p* values indicate a significance difference between these two groups.

## Data Availability

Data presented in this study are available upon request from the corresponding authors.
